# CD133 Expression in the Nucleus Is Associated with Endometrial Carcinoma Staging and Tumor Angioinvasion

**DOI:** 10.3390/jcm10102144

**Published:** 2021-05-15

**Authors:** Milosz Pietrus, Kazimierz Pitynski, Marcin Waligora, Katarzyna Milian-Ciesielska, Monika Bialon, Artur Ludwin, Klaudia Skrzypek

**Affiliations:** 1 Department of Gynecology and Oncology, Faculty of Medicine, Jagiellonian University Medical College, 31-501 Krakow, Poland; milosz.pietrus@uj.edu.pl (M.P.); kazimierz.pitynski@uj.edu.pl (K.P.); mbialon524@gmail.com (M.B.); ludwin@cm-uj.krakow.pl (A.L.); 2 Center for Innovative Medical Education, Department of Medical Education, Faculty of Medicine, Jagiellonian University Medical College, 30-688 Krakow, Poland; marcin.waligoora@gmail.com; 3 Department of Pathomorphology, Faculty of Medicine, Jagiellonian University Medical College, 31-531 Krakow, Poland; kmilianciesielska@gmail.com; 4 Institute of Pediatrics, Department of Transplantation, Faculty of Medicine, Jagiellonian University Medical College, 30-663 Krakow, Poland

**Keywords:** CD133 (PROM1), endometrial carcinoma, tumor metastasis, angioinvasion, tumor grading, patient prognosis

## Abstract

Background: (1) Endometrial cancer is one of the most common cancers affecting women, with a growing incidence. To better understand the different behaviors associated with endometrial cancer, it is necessary to understand the changes that occur at a molecular level. CD133 is one of the factors that regulate tumor progression, which is primarily known as the transmembrane glycoprotein associated with tumor progression or cancer stem cells. The aim of our study was to assess the impact of subcellular CD133 expression on the clinical course of endometrial cancer. (2) Methods: CD133 expression in the plasma membrane, nucleus, and cytoplasm was assessed by immunohistochemical staining in a group of 64 patients with endometrial cancer representing FIGO I-IV stages, grades 1–3 and accounting for tumor angioinvasion. (3) Results: Nuclear localization of CD133 expression was increased in FIGO IB-IV stages compared to FIGO IA. Furthermore, CD133 expression in the nucleus and plasma membrane is positively and negatively associated with a higher grade of endometrial cancer and angioinvasion, respectively. (4) Conclusions: Our findings suggest that positive nuclear CD133 expression in the tumor may be related to a less favorable prognosis of endometrial carcinoma patients and has emerged as a useful biomarker of a high-risk group.

## 1. Introduction

CD133 is a transmembrane glycoprotein with multiple N-glycan structures that are important in tumor progression. It is encoded by the prominin 1 (PROM1) gene and is glycosylated following transcription and translation [1]. CD133 is typically localized in plasma membrane protrusions and microvilli, suggesting a role in membrane organization [2]. Nevertheless, its subcellular organization may be involved in different signaling pathways, as it may bind to cholesterol-containing lipid rafts [3].

Many studies suggest that the level of CD133 expression is correlated with cancer patient prognosis and survival [4]. Furthermore, its role in maintaining stem cell-like properties has been suggested [5]. Recently, the nuclear localization of its expression in tumors has also been widely described in the literature [6,7]. Atypical nuclear localization of the membrane proteins, including CD133, can characterize cellular plasticity in colorectal cancer progression, represents mesenchymal phenotype, and characterizes cell plasticity in cancer progression [8]. It was suggested that CD133 in the nucleus can function as a transcriptional regulator via interfering with the molecular pathways associated with proliferation and differentiation [9,10]. In hepatocellular carcinoma, nuclear localization may also play a role in rescue of high expression levels, when it is highly expressed in the cytoplasm during tumor progression [11].

CD133 may also be an important factor in endometrial cancer, which is one of the most common cancers in women [12]. The prognosis of endometrial adenocarcinoma is strongly associated with advanced malignancy and disease staging [13], especially for grade 3 [14]. Similarly, more advanced local disease spread according to FIGO implies a high disease risk and worse prognosis [15]. Survival is increased in patients who are younger, have early stage disease, and have lower-grade disease [13]. Moreover, endometrial carcinoma incidence tends to increase, most likely due to the prolongation of the women’s lives and various environmental factors. This tumor responds well to different oncological treatments; however, disease relapse and spread occurs in 15–20% of cases [16]. Understanding the changes that occur at the molecular level will help to better understand this phenomenon.

CD133 may be one of the factors crucial in endometrial cancer progression; however, its role remains to be verified in different cellular compartments. Interestingly, it was shown that its promoter may be hypomethylated in malignant endometrial cancer [17]. The upregulated expression was also detected in the Ishikawa human endometrial adenocarcinoma cell line [18]. There are studies suggesting that CD133+CXCR4+ cells may possess some characteristics of CSCs in primary endometrial cancer [19]. Nevertheless, the literature also contains contradictory data. One paper suggests that the CD133+ tumor status correlates with a favorable prognosis of endometrial cancer and a lower rate of vascular invasion and higher differentiation status [20]. In contrast, another paper showed that CD133+ cells express more cancer stem-cell markers and exhibit an enhanced proliferation rate [21,22], resistance to chemotherapeutics [22,23], and enhanced tumorigenicity in immunodeficient mice in vivo [22,23]. Due to contradictory data and a lack of information regarding the nuclear localization, its role remains to be further studied in endometrial cancer.

In this study, we evaluated the importance of CD133 expression in endometrial carcinoma and its correlation between grade, stage, and cellular localization.

## 2. Materials and Methods

### 2.1. Patients and Ethics Approval

Our manuscript conforms to the Enhancing the QUAlity and Transparency Of health Research (EQUATOR) network guidelines.

The study was approved by the Bioethics Committee of the Jagiellonian University in Krakow, Poland (number 1072.6120.223.2017) from 30 November 2017. It was planned to retrospectively analyze 25 consecutive patients for each grading groups: 1, 2, and 3 (for a total of 75), that received optimal treatment that included an operation for endometrial cancer at the Gynecology and Oncology Unit of the University Hospital in Krakow in years 2010–2016. All diagnostic and therapeutic procedures were performed according to the current European Society for Gynecological Oncology (ESGO) guidelines. The archival material in the form of paraffin blocks stored at the Pathomorphology Department of the Jagiellonian University was used in this study. The inclusion criteria confirmed the diagnosis of endometrial cancer, proper quality of the archival material, no prior neoadjuvant treatment, and complete medical documentation. Staging according to the FIGO classification was based on the surgical protocol and results of a pathomorphological examination.

### 2.2. Immunohistochemical Analysis of CD133 Expression

We assessed the level of CD133 expression by immunohistochemical staining (IHC) in 64 FFFPE primary human endometrial cancer specimens. Two board-certified pathologists evaluated the hematoxylin and eosin (H&E) stained slides for all of the patients to make a final diagnosis and provide pathological staging of the disease according to FIGO classification. All the slides were evaluated by two board-certified histopathologists. Immunohistochemistry was performed on tissue sections (3-μm thick). The slides were deparaffinized, rehydrated in 100% ethanol, immersed in 3% H_2_O_2_ at room temperature (RT) to block endogenous peroxidase, and washed in distilled water, as well as wash buffer (Tris/HCl, DakoCytomation, S3006). To unmask the antigen, the slides were microwaved in an antigen-retrieval solution (EDTA buffer, pH 8.0), after which a blocking solution was applied (Ultra Vision Protein Block) in a humidified chamber for 5 min at room temperature. Primary antibodies were applied to each tissue section and incubated for 30 min in a humidified chamber. The primary anti-CD133 rabbit polyclonal antibody 70R-13813 (Fitzgerald, Acton, MA, USA) was used at a 1:50 dilution. Next, the slides were washed in wash buffer and incubated for 20 min with BrightVision+ Goat anti-mouse/rabbit HRP. The enzymatic reaction was performed with a DAB incubation for 3–8 min at RT. Tissue sections were counterstained with hematoxylin and placed under a coverslip. The sections were washed in distilled water and cooled at room temperature (RT) for 20–30 min. For the positive control, the same method was used in human breast-cancer tissue. For the negative control, the same specimen and method were used without the primary antibody. In each endometrial cancer specimen, the representative in terms of quality of staining and viability of tumor (at least 75% of viable cancer cells) high-powered field (HPF) (400×) of tumor area was chosen for further examination. The percentage of cells exhibiting cytoplasmic, nuclear, and a membranous staining pattern was evaluated only in the tumor area. Additionally, in each case in which angioinvasion was present, the cancer cells inside the vessels were assessed. All evaluations were performed blindly by two board-certified histopathologists. In case of disagreements in scoring, the slides were reviewed until a consensus was obtained, which occurred in three cases. Calculations were performed using a ZEISS AXIO Lab.A1 microscope. Representative images were collected using an Olympus SC180 digital camera.

### 2.3. Bioinformatical and Statistical Analysis of the Data

A statistical analysis was performed using Statistica PL software [StatSoft, Inc. (2010); STATISTICA (data analysis software system), version 12.0, Tulsa, OK 74104, USA www.statsoft.com (accessed on March 2021)] and MedCalc Statistical Software version 16.8 (MedCalc Software bvba, Ostend, Belgium; https://www.medcalc.org (accessed on March 2021); 2016). Continuous variables were reported using the means and standard deviations. Categorical variables are described as counts and percentages. Continuous variables were compared using a Mann–Whitney U-test. An χ^2^-test was used to compare the categorical variables. A Friedman test was used to compare the expression of CD133 in the tumor grading group (1–3) with a post hoc analysis when there was a significant difference. The relationship between the percentage of cells expressing CD133 and increasing grading stages was estimated using a Spearman test. A series of multiple logistic regression models was used to assess the association in the percentage of CD133+ expression in different compartments (nucleus, cytoplasm, and membrane), as well as tumor grading (1 and 2 vs. 3) or staging (Ia vs. Ib-IV according to FIGO). The variables were selected using forward selection and were included if *p* < 0.2. The determinants of malignancy that were significant in the logistic regression models were further used in receiver-operating characteristic analyses (ROC) with classification variables, including tumor grading (1 and 2 vs. 3) and staging (IA vs. IB-IV according to FIGO). For each ROC analysis, the area under the curve (AUC) and associated 95% confidence intervals (CI) were calculated. Additionally, the cut-off values with the highest level of sensitivity and specificity were established.

## 3. Results

### 3.1. CD133 Is Positively Expressed in the Nucleus, Whereas Its Expression in the Plasma Membrane Is Negatively Associated with Higher Grade and Stage of Endometrial Cancer

In this study, out of 75 initially identified consecutive cases of endometrial carcinoma FIGO Stage I to IV and grade of 1 to 3, 11 patients did not meet the inclusion criteria, met exclusion criteria or had unsatisfactory quality of histological material for analysis. The characteristics of eligible patients are summarized in Table 1. The mean age in the study group was 61.2 ± 11.3 years. The average menstrual cycle length was 28.7 ± 3.4 days and menstruation lasted for 5.5 ± 3.3 days. Nevertheless, most of the patients developed endometrial cancer after menopause. The mean age at the first menstruation was 13.8 ± 1.5 years, whereas the mean age at the last menstruation was 50.7 ± 4.3 years.

**Figure 1:**
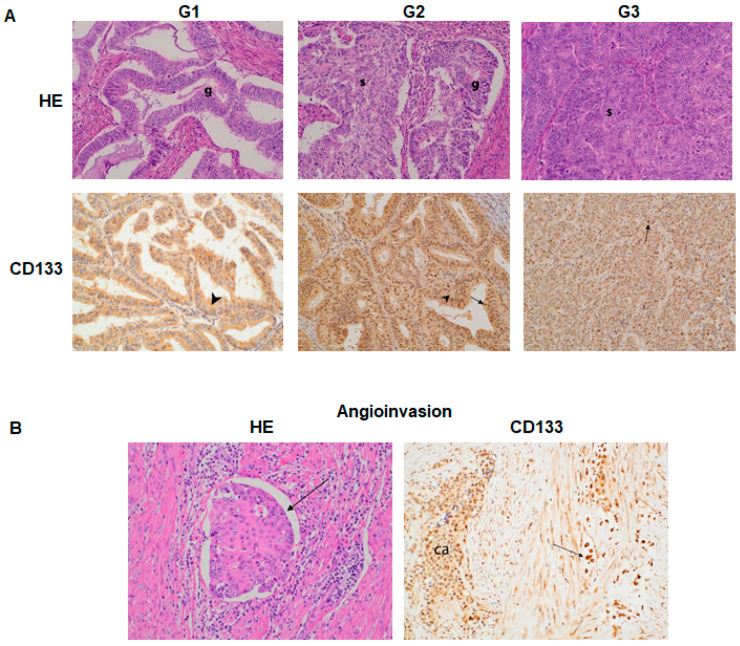
The level of CD133 expression in endometrial carcinoma as evaluated by immunohistochemical staining. (**A**) The level of CD133 expression in tumors displaying different grading. G1, well-differentiated (G1) endometrial adenocarcinoma exhibiting exclusively glandular pattern (g). HE, hematoxylin-eosin staining; CD 133 stain, cytoplasmic pattern (arrowhead); magnification 200×. G2, moderately differentiated (G2) endometrial adenocarcinoma with glandular (g) and solid (s) areas; CD133 staining, cytoplasmic pattern (arrowhead) and nuclear pattern (arrow); magnification 200×. G3, poorly differentiated (G3) endometrial adenocarcinoma exhibiting almost an exclusively solid pattern (s); HE, magnification 200×; CD133 stain, cytoplasmic and nuclear (arrow) pattern in almost all cells; magnification 100×. (**B**) Poorly differentiated (G3) endometrial adenocarcinoma, angioinvasion-cancer cells inside the lymphatic vessel (arrow); HE stain, magnification 400×; poorly differentiated (G3) endometrial adenocarcinoma (ca), angioinvasive component inside the lymphatic vessel exhibiting nuclear staining (arrow); CD133 staining; magnification 200×.

The 64 patients with endometrial carcinoma were included in the analysis of the level of CD133 expression. Tumor grade staging was based on hematoxylin-eosin staining. The presence of CD133 was evaluated in the plasma membrane, cytoplasm, and nucleus of the tumor cells (Figure 1A), as well as the angioinvasive parts (Figure 1B) by immunohistochemical staining.

The level of CD133 expression in the cellular compartments was associated with the tumor grade as presented in Table 2. There were no differences in the percentage of cells exhibiting cytoplasmic CD133 expression between the different grades. Nevertheless, the percentage of tumor cells exhibiting nuclear localization of CD133 was increased in higher graded stages, whereas expression in the plasma membrane was decreased. The results are summarized in Table 2.

We found that the percentage of cells displaying expression in the plasma membrane was negatively correlated with increased malignancy as measured by grading (R = −0.46, *p* < 0.001). In contrast, an increased count of CD133+ cells in the nucleus was positively correlated with tumor grading (R = 0.57, *p* < 0.001). There was no such relationship observed in the case of CD133 expression in the cytoplasm (R = −0.06, *p* = 0.65).

Furthermore, tumor grading was determined by both the percentage of cells with CD133 expression in the nucleus and membrane as calculated in the logistic regression analysis (*p* = 0.0004; R^2^ = 0.32). For every 1% of more cells expressing CD133 in the nucleus, there was a 3% higher chance that the cancer grade would be 3 (OR = 1.03; 95% Cl = 1.001–1.05, *p* = 0.004). Accordingly, for every 1% of cells expressing CD133 in the plasma membrane there was a 3% lower chance that the cancer grade would be 3, rather than grades 1 or 2 (OR = 0.97; 95% Cl = 0.95–0.996, *p* = 0.02).

**Figure 2:**
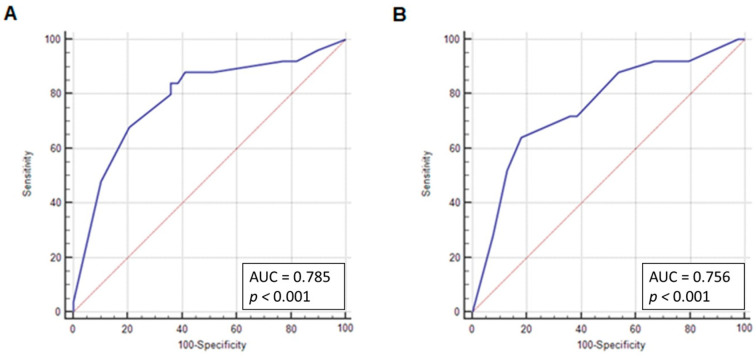
ROC curves for determining CD133 expression cut-off value in grading and FIGO staging. (**A**) ROC curve for determining CD133 nuclear localization cut-off value in grade 3. Cut-off point > 50%, *p* < 0.001, AUC = 0.79, sensitivity = 84%, and specificity = 64.1%. (**B**) ROC curve for determining the localization of CD133 in the plasma membrane cut-off value in grade 3. Cut-off point ≤ 10%, *p* < 0.001, AUC = 0.76, sensitivity = 64%, specificity = 82.1%.

ROC curves were drawn to determine the sensitivity and specificity of CD133 expression in the nucleus and plasma membrane to be associated with a stage of grade 3. For nuclear expression, the area under the curve (AUC) was 0.79. The best cut-off point for CD133 nuclear expression was > 50% with a sensitivity of 84% and a specificity of 64.1% (Figure 2A). Accordingly, for expression in the plasma membrane, the AUC was 0.76 and the best cut-off point for CD133 expression in the plasma membrane was established for ≤ 10% with a sensitivity of 64% and a specificity of 82.1% (Figure 2B).

### 3.2. CD133 Expression in the Nucleus Is Positively Associated with Higher FIGO Stages IB-IV in Comparison to FIGO IA

Expression of CD133 in the cellular compartments was also compared in FIGO IB-IV stages to FIGO IA. Similar to the grading results, the percentage of cells with nuclear localization was increased in the higher FIGO stages (FIGO IB-IV) compared to FIGO IA (Table 3).

The percentage of tumor cells expressing CD133 expression in the plasma membrane tended to be slightly negatively correlated with the increasing FIGO stages (R = −0.22, *p* = 0.13), whereas the nucleus tended to be slightly positively correlated with the increased FIGO stages (R = −0.22, *p* = 0.08). Nevertheless, there was no effect for CD133 expression in the cytoplasm (R = 0.08, *p* = 0.5).

Furthermore, the logistic regression (*p* = 0.006, R^2^ = 0.15) showed that for every 1% or more cells in the nucleus expressing CD133, there was a 2% higher chance that the cancer stage will be at least IB, and not IA according to FIGO (OR = 1.02; 95% Cl = 1.006–1.04, *p* = 0.009). The regression model did not include insignificant variables, such as membrane or cytoplasmic CD133 expression.

**Figure 3:**
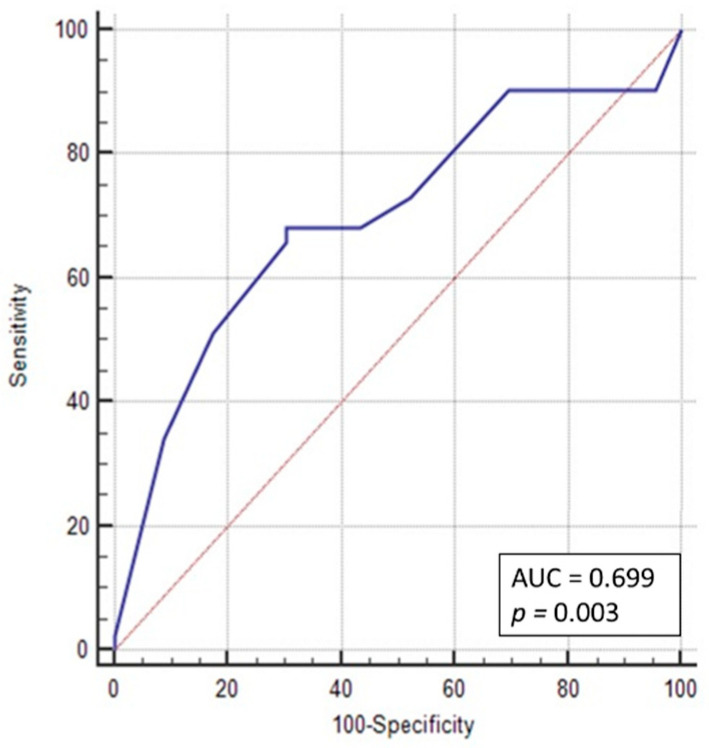
ROC curve for determining the nuclear CD133 cut-off value in FIGO staging. Cut-off point > 50%; *p* = 0.003; AUC = 0.7; sensitivity = 68.3%; specificity = 69.6%.

An ROC curve was drawn to determine the sensitivity and specificity of nuclear CD133 expression to be associated with the FIGO IB-IV disease stages. The area under the curve was 0.7. The best cut-off point for CD133 nuclear expression was 50%, with a sensitivity of 68.3%% and a specificity of 69.6% (Figure 3).

### 3.3. CD133 Expression in the Nucleus and Plasma Membrane Is Positively and Negatively Associated with Tumor Angioinvasion, Respectively

Since tumor cell invasion into the vasculature is a crucial feature in tumor progression, the level of cellular CD133 expression was also associated with the appearance of tumor cell angioinvasion. The logistic regression model (*p* = 0.0004, R^2^ = 0.32) revealed that the independent determinants of angioinvasion were nuclear and membrane CD133 expression. For every 1% of the cells expressing CD133 in the nucleus, there was a 2% higher chance of finding tumor angioinvasion (OR = 1.02; 95% Cl = 1.005–1.047; *p* = 0.04). Accordingly, for every 1% of cells expressing CD133 in the plasma membrane, there was a 3% lower chance of finding tumor angioinvasion (OR = 0.97; 95% Cl = 0.94–0.998; *p* = 0.03).

**Figure 4:**
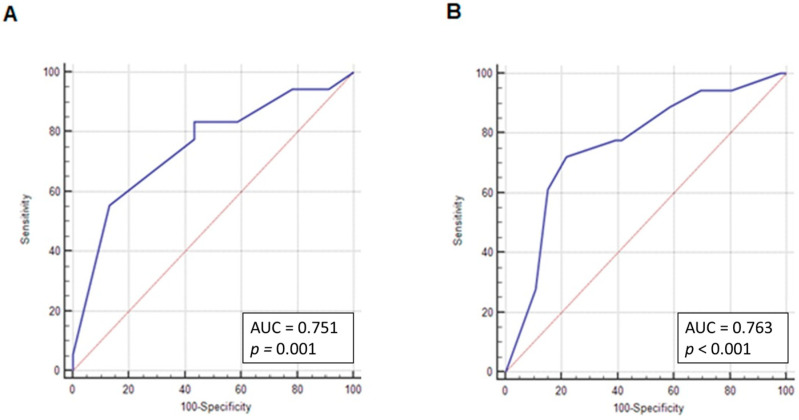
ROC curves for determining the CD133 cut-off value in tumor angioinvasion. (**A**) ROC curve for determining the CD133 nuclear localization cut-off value in tumor angioinvasion. Cut-off point > 80%; *p* = 0.001; AUC = 0.75; sensitivity = 55.6%; specificity = 87%. (**B**) ROC curve for determining CD133 localization in the plasma membrane cut-off value in tumor angioinvasion. Cut-off point ≤ 10%; *p* < 0.001; AUC = 0.76; sensitivity = 72.2%; specificity = 78.3%.

ROC curves were drawn to determine the sensitivity and specificity of CD133 expression in the nucleus and plasma membrane to predict angioinvasion of tumor cells. The area under the curve was 0.75 for nuclear expression. The best cut-off point for CD133 nuclear expression was > 80%, with a sensitivity of 55.6% and specificity of 87% (Figure 4A). Accordingly, for expression in plasma membrane, the AUC was 0.76 with the best cut-off point for CD133 expression ≤ 10% (sensitivity of 72.2% and a specificity of 78.3%) as shown in Figure 4B.

## 4. Discussion

These results indicate that the nuclear expression of CD133 may predict advanced malignancy for patients with endometrial cancer, as CD133 expression in the nucleus and plasma membrane is positively and negatively associated, respectively, with a higher grade and FIGO stages, as well as more intense angioinvasion.

In endometrial cancer, the correlation between the level of CD133 expression and patient prognosis were described regarding its localization in the plasma membrane. CD133 is a transmembrane glycoprotein with multiple N-glycan structures [1] that has been proposed to be a marker for the identification and isolation of endometrial CSCs [5,23,24]. CD133-positive cells purified from endometrial adenocarcinomas displayed a higher level of proteins associated with tumor progression (e.g., matrix metalloproteases, interleukin-8, CD44, and CXCR4) [23]. Furthermore, CD133 together with other markers (e.g., CD44), were used to isolate and enrich carcinoma-initiating cells (CICs) from endometrial carcinoma cell lines, and those cells were also more resistant to the inhibition of growth when treated with cisplatin and paclitaxel, and formed tumors more easily in vivo [25]. Nevertheless, another study suggested that CD44 and CD133 may be involved in the early stages of endometrial cancer development, but not the late ones, since CD133 expression is higher during the early tumor stage (FIGO I-II) compared with those of FIGO III to IV stage disease [26].

Thus, the literature consists of contradictory data. CD133 has been described to be associated with either a more [20] or less favorable prognosis [22] for the patients, or its role may depend on tumor stages [26]. The missing factor in previous research into endometrial cancer is its subcellular localization. Our results suggest that CD133 localization in the different cellular compartments may clarify its prognostic value. CD133 subcellular organization may be involved in the different signaling pathways. For example, CD133 may bind to cholesterol-containing lipid rafts [3], whereas it can act as a transcriptional regulator following translocation to the nucleus via interfering with the molecular pathways associated with proliferation and differentiation [9,10].

Grade 1 or 2 tumors had equal disease-specific survival rates (92% and 94% 5-year survival, respectively), whereas patients with Grade 3 tumors had a significantly worse outcome (63% 5-year survival). These findings indicate that from a clinical perspective, discrimination between Grade 1 and Grade 2 is not very useful [14]. The percentage of 5-year survival for clinical degrees using the current FIGO scale was estimated as follows: in stage I: IA—89.2%, IB—75.1%; in stage II: 75–87.3%; in stage III: IIIA—76%-83.8%, III B—60–65%, IIIC—61.3–75.5%; and grade IV: 17–27% [27]. However, patients with stage IA G1/G2 endometrial cancer are included in the low-risk group; therefore, they do not require adjuvant treatment. In our study, we decided to compare the groups of patients in stage IA vs. IB + with grading G1, G2 vs. G3. This approach showed that an assessment of CD133 expression can provide early information regarding tumor grading and staging. This finding highlights a possible clinical approach to the expected malignant type of disease in particular patients.

In different tumor types, the cellular localization of CD133 turned out to be an important factor of tumor prognosis. Its nuclear localization indicated a poor prognosis or increased tumor progression in hepatocellular carcinoma [6], colorectal cancer [8], and triple negative breast cancer [9].

Nuclear CD133 may either play the role of rescue for high cytoplasmic CD133 expression during tumor progression [11], or it may function as a transcriptional regulator [9,10]. A second role may explain the association of nuclear localization with higher disease stages of endometrial cancer. CD133 expression in the nucleus may interfere with the molecular pathways associated with tumor proliferation or differentiation [9,10] and this role may be more important for tumor progression than its role in the plasma membrane. In murine cells, the nuclear translocation of CD133 together with ciliary membrane components was particularly evident in transit amplifying cells and immediate stem-cell derivatives. The molecular mechanism of CD133 in the nucleus indicates that Glis2 is a partner of CD133 in the nucleus and mediates activation of SHH signaling. The CD133-Glis2 complex is imported to the nucleus in a Ran-GTP manner, and the Stat3 transcription factor is subsequently a direct downstream target of CD133-Glis2 signaling [28]. A similar mechanism of STAT3 activation by nuclear CD133 may also be responsible for increased tumor progression in endometrial cancer, since STAT3 was previously shown to be activated in human endometrial cancers and inhibition of its signaling was suggested to be an effective target for cancer treatment [29]. Similarly, enhanced SHH signaling may be associated with the progression of endometrial cancer. Abnormal activation of this pathway was previously described to be involved in the proliferation of endometrial carcinoma cells [30]. Moreover, the aberrant expression of key components of the SHH signaling pathway may serve as a prognostic factor for recurrence and survival in patients with endometrial cancer [31]. Nevertheless, the unexpected function of nuclear CD133 expression in endometrial carcinoma requires future study.

Our results are in agreement with those of previous studies, which investigated only the cell surface expression of CD133 and are thus correlated with the CD133+ tumor status. A favorable prognosis of endometrial cancer was associated with a lower rate of vascular invasion and higher differentiation status [20]. Our findings indicate that CD133 expression in the plasma membrane and nucleus is both negatively and positively associated with tumor angioinvasion, respectively. Development of new blood vessels inside the tumor and invasion of tumor cells inside the vasculature are good indicators of tumor progression and its subsequent metastasis [32].

We believe that our study has several strengths. First, it is a novelty that consists of new insights of CD133 in different cellular compartments and clinical impact that links immunohistochemical findings with cancer stage and grade. Second, we provided its possible pathomechanism that included histological evidence of angioinvasion, which was also attributed to higher CD133 nuclear localization.

On the other hand, our study also has drawbacks. The main limitation of our study is its single center and retrospective approach, which resulted from its exploratory design. We have observed different cancer stages in grading groups 1, 2, and 3. However, this is due consecutive enrollment of patients. In the future, our results should be validated in a higher number of patients with more than one method.

The patients who were included in our study underwent standard procedures, consisting mainly of total hysterectomy with bilateral salpingooophorectomy and, in most cases, total pelvic lymphadenectomy, optionally with para-aortic lymphadenectomy, in line with ESGO guidelines at that time. One of the factors determining the extent of the procedure was belonging to the risk group. However, recently a new recommendation ESMO/ESTRO/ESP was published, as well as other reports, suggesting the use of the Sentinel lymph node biopsy procedure can be considered for staging purposes in patients with low-risk or intermediate-risk disease [33,34,35].

## 5. Conclusions

In summary, our results showed that increased levels of CD133 expression in the nucleus and its lower level in plasma membrane are associated with a higher endometrial carcinoma disease stage and tumor angioinvasion. Thus, the nuclear CD133+ tumor status may be correlated with a less favorable prognosis of endometrial carcinoma patients. Therefore, our results suggest that the CD133 tumor status may emerge as a useful biomarker of low- or high-risk endometrial carcinoma, and it may help to plan treatment therapies for patients in the future. It may become a predictive factor for including more radical and aggressive adjuvant therapy. Our results suggest that a higher nuclear and lower membrane expression of CD133 in endometrial cancer cells may be a positive predictor of a worse prognosis in patients. Hence, there may be a need for more aggressive basic or adjuvant therapy. Nevertheless, more studies focused on CD133 role in treatment strategies are necessary in future.

## Figures and Tables

**Table 1:** Characteristics of the patients and endometrial carcinoma staging.

Number of Patients	64
Age	61.2 ± 11.3
Age of the first menstruation	13.8 ± 1.5
Length of women’s monthly cycle (days)	28.7 ± 3.4
Length of menstruation (days)	5.5 ± 3.3
Age of the last menstruation	50.7 ± 4.3
Number of pregnancies	
0	4 (6.3%)
1	13 (20.3%)
2	27 (42.2%)
3	14(21.9%)
4	4 (6.3%)
5	0
6	1 (1.6%)
Lack of data	1 (1.6%)
FIGO	
I	43 (67.2%)
II	12 (18.8%)
III	8 (12.5%)
IV	1 (1.6%)
Grading	
1	17 (26.6%)
2	23 (35.95%)
3	24 (37.5%)

**Table 2:** Cellular localization of CD133 is associated with endometrial cancer grading. Legend: * *p* < 0.0001, post hoc analysis in comparison to the level of nuclear CD133 expression in grade 1. † < 0.0001, post hoc analysis in comparison to the level of nuclear CD133 expression in grade 2. ** *p* = 0.01, post hoc analysis in comparison to CD133 expression in the plasma membrane in grade 2. ‡ *p* < 0.0001, post hoc analysis in comparison to the expression of CD133 in the plasma membrane in grade 1.

	Grade 1 (*n* = 17)	Grade 2 (*n* = 23)	Grade 3 (*n* = 24)	*p*-Value
CD133 in cytoplasm (% of cells)	85 ± 16.1	80.4 ± 16.2	83.5 ± 10.6	0.61
CD133 in nucleus (% of cells)	27.9 ± 31.3	47 ± 30	75.7 ± 23.8 * †	<0.0001
CD133 in plasma membrane (% of cells)	48.8 ± 25	36.4 ± 29.5	16.1 ± 22.3 ** ‡	0.005
FIGO				
I	15 (88.2%)	18 (78.3%)	10 (41.7%)	
II	1(5.9%)	4 (17.4%)	7 (29.2%)	
III	1 (5.9%)	1 (4.4%)	6 (25%)	0.04
IV	0	0	1 (4.2%)	

**Table 3:** Nuclear localization of CD133 is increased in FIGO IB-IV stages compared to FIGO IA.

	FIGO IA (*n* = 23)	FIGO IB-IV (*n* = 41)	*p*-Value
CD133 in cytoplasm (% of cells)	82.6 ± 12.5	82.3 ± 15.2	0.94
CD133 in nucleus (% of cells)	37.4 ± 30.8	61.2 ± 32.9	0.006
CD133 in plasma membrane (% of cells)	38.6 ± 29.1	28.5 ± 28.2	0.18

## Data Availability

Data is contained within the article.
